# A nomogram based on the quantitative and qualitative features of CT imaging for the prediction of the invasiveness of ground glass nodules in lung adenocarcinoma

**DOI:** 10.1186/s12885-024-12207-8

**Published:** 2024-04-09

**Authors:** Yantao Yang, Jing Xu, Wei Wang, Mingsheng Ma, Qiubo Huang, Chen Zhou, Jie Zhao, Yaowu Duan, Jia Luo, Jiezhi Jiang, Lianhua Ye

**Affiliations:** 1 Department of Thoracic and Cardiovascular Surgery, Yunnan Cancer Hospital, the Third Affiliated Hospital of Kunming Medical University, No. 519 Kunzhou Road, Xishan District, Yunnan Province, Kunming, China; 2 Department of Dermatology and Venereal Diseases, Yan’an Hospital of Kunming City, Kunming, China; 3 Department of Thoracic and Cardiovascular Surgery, Shiyan Taihe Hospital (Hubei University of Medicine), Hubei, Shiyan, China; 4 Department of Pathology, Yunnan Cancer Hospital, the Third Affiliated Hospital of Kunming Medical University, Kunming, China; 5 Department of Radiology, Yunnan Cancer Hospital, the Third Affiliated Hospital of Kunming Medical University, Kunming, China

**Keywords:** Ground glass nodule, Radiologic characteristic, Lung adenocarcinoma, Invasiveness, Prediction model, Nomogram

## Abstract

**Purpose:**

Based on the quantitative and qualitative features of CT imaging, a model for predicting the invasiveness of ground-glass nodules (GGNs) was constructed, which could provide a reference value for preoperative planning of GGN patients.

**Materials and methods:**

Altogether, 702 patients with GGNs (including 748 GGNs) were included in this study. The GGNs operated between September 2020 and July 2022 were classified into the training group (*n* = 555), and those operated between August 2022 and November 2022 were classified into the validation group (*n* = 193). Clinical data and the quantitative and qualitative features of CT imaging were harvested from these patients. In the training group, the quantitative and qualitative characteristics in CT imaging of GGNs were analyzed by using performing univariate and multivariate logistic regression analyses, followed by constructing a nomogram prediction model. The differentiation, calibration, and clinical practicability in both the training and validation groups were assessed by the nomogram models.

**Results:**

In the training group, multivariate logistic regression analysis disclosed that the maximum diameter (OR = 4.707, 95%CI: 2.06–10.758), consolidation/tumor ratio (CTR) (OR = 1.027, 95%CI: 1.011–1.043), maximum CT value (OR = 1.025, 95%CI: 1.004–1.047), mean CT value (OR = 1.035, 95%CI: 1.008–1.063; *P* = 0.012), spiculation sign (OR = 2.055, 95%CI: 1.148–3.679), and vascular convergence sign (OR = 2.508, 95%CI: 1.345–4.676) were independent risk parameters for invasive adenocarcinoma. Based on these findings, we established a nomogram model for predicting the invasiveness of GGN, and the AUC was 0.910 (95%CI: 0.885–0.934) and 0.902 (95%CI: 0.859–0.944) in the training group and the validation group, respectively. The internal validation of the Bootstrap method showed an AUC value of 0.905, indicating a good differentiation of the model. Hosmer–Lemeshow goodness of fit test for the training and validation groups indicated that the model had a good fitting effect (*P* > 0.05). Furthermore, the calibration curve and decision analysis curve of the training and validation groups reflected that the model had a good calibration degree and clinical practicability.

**Conclusion:**

Combined with the quantitative and qualitative features of CT imaging, a nomogram prediction model can be created to forecast the invasiveness of GGNs. This model has good prediction efficacy for the invasiveness of GGNs and can provide help for the clinical management and decision-making of GGNs.

## Introduction

According to the Global Cancer Report 2020, lung cancer remains the dominant reason for tumor death, and the most common histological subtype is adenocarcinoma [1]. Lung adenocarcinoma was classified as [2] adenocarcinoma in situ (AIS), atypical adenomatous hyperplasia (AAH), minimally invasive adenocarcinoma (MIA), as well as invasive adenocarcinoma (IAC) based on the World Health Organization (WHO) in 2015. With the widespread application of high-resolution computed tomography (CT), more and more lung adenocarcinomas have been found to be manifested with ground glass nodules (GGNs). GGN refers to the focal lung tissue presenting a cloud with increased density on high-resolution CT, whereas the normal internal structure can still be seen. Generally, GGNs can be allocated into pure GGN (pGGN) and part solid nodules (PSN) [3] according to whether they contain solid components. Lung adenocarcinoma with GGNs can be pathologically diagnosed as AIS, MIA, or IAC. Especially, AIS and MIA are known as non-invasive adenocarcinoma (NIAC), and patients with NIAC have 100% 10-year disease-free survival (DFS) after complete resection [4]. However, in IAC with pathological stage IA, the 5-year DFS was only 89.0% [5]. The prognosis of patients varies greatly with the size of the tumor invasion. Accurate identification of NIAC is essential for choosing the timing of surgery and achieving an excellent prognosis.

In the tissue specimens obtained from intraoperative frozen sections, there are obvious limitations in judging the size of tumor invasion [6]. In comparison, preoperative evaluation of the size of lung adenocarcinoma with CT features exhibits obvious advantages. Previous articles have revealed that [7, 8] CT features can identify the invasiveness of GGN, but different studies have different parameters for identifying the invasiveness of GGN. Fu et al. [7] believed that maximum diameter was the only effective indicator to judge the invasiveness of GGN. A meta-analysis suggested that qualitative CT features exerted a limited function in the differentiation of invasive GGNs, with a diagnostic sensitivity of 0.41–0.52 and a specificity of 0.56–0.63 [9]. However, He [10] believed that CT qualitative features also produced a marked effect in the invasive identification of GGN. He et al. [11] conducted a meta-analysis involving 8 papers and concluded that the mean CT value could also identify the invasiveness of GGN, with a specificity of 0.81 and a sensitivity of 0.78. Recently, some scholars believe that the maximum CT value [12] can also predict the infiltration of GGN. These studies mostly focused on the relationship between a single imaging feature and the infiltration of GGN.

Some scholars have discussed whether the combination of multiple features can improve the diagnostic efficacy. Li et al. [13] concluded in a study involving 216 GGNs that the diagnostic model combined with the quantitative and qualitative features of CT could maximize diagnostic efficiency with an AUC value of 0.931. Yan et al. [14] obtained similar results by using the same method. Liu et al. [15] compared the models of CT quantitative features, CT qualitative features, and CT quantitative in a combination with qualitative features in a study containing 160 GGNs, and the AUC values were 0.803, 0.854, and 0.873, respectively. However, few studies have verified the model after its establishment, which limits its practical application in the clinic. Moreover, each study screened out the quantitative and qualitative features of CT on the basis of small sample size, and the results were different. The optimal CT parameters for model construction still need to be systematically discussed on the basis of large samples. In addition, the nomogram can visualize the equations and make the results more practical and readable compared with previous studies which include complex prediction equations. Based on this, we plan to conduct a large-sample study to systematically explore the relationship between the quantitative and qualitative features of CT imaging and the invasiveness of GGN, search for independent predictors, construct and verify the nomogram model for predicting the risk of IAC, and provide a basis for the selection of clinical treatment timing and methods of GGN.

## Materials and methods

### Participants

**Fig. 1:**
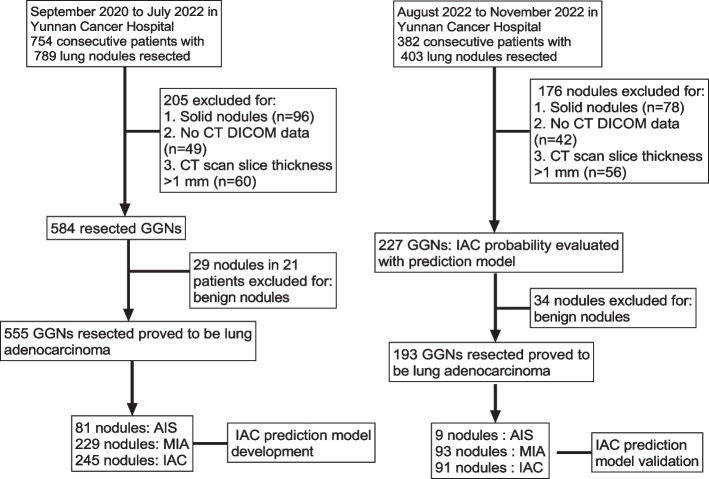
Patient screening flowchart

This study got approval from the ethics committee of our hospital (KYLX2022206), which exempted the requirement of informed consent of patients in this retrospective study. Clinical data and chest CT images were retrospectively collected from patients with GGN which resected in the Third Affiliated Hospital of Kunming Medical University from September 2020 to November 2022. Inclusion criteria: (1) All patients had CT image data of our hospital within 2 weeks before surgery, with one or more GGNs; (2) After surgical resection of all patients, the GGNs were pathologically diagnosed as lung adenocarcinoma (including AIS, MIA, and IAC); (3) There were one or more GGNs for surgical intervention; (4) None of the patients underwent anti-tumor therapy such as radiotherapy and chemotherapy before surgery; (5) patients aged ≥ 18 years old. Exclusion criteria: (1) Patients with incomplete imaging data or medical records; (2) Patients with lung infection causing image interference with imaging analysis conditions; (3) The large respiratory movement artifact in the image did not meet the requirements of imaging analysis; (4) Unclear correlation between the position of GGNs in the postoperative pathological reports and those in the preoperative CT images. The patients were classified into two parts following the time of operation: (1) 555 GGNs from September 2020 to July 2022 were used as the training group; (2) 193 GGNs from August 2022 to November 2022 were used as the validation group (Fig. 1).

### CT acquisition

Before the examination, patients were given breathing training. During the scan, patients were placed in a supine position with the arms raised, holding their breath after a deep inspiration or after a calm breath. Using the siemens 64-row 128-slice spiral CT machine, a spiral scanning was performed from the lung tip to the bottom of the lung with a tube voltage of 120 kV, current of 100 mAs, pitch of 1.0, and layer thickness of 1 mm. Reconstruction parameters: 512 × 512 matrix. High resolution lung algorithm, Lung window: 1200 to 1500HU window width, -600 to -700HU window position, Standard soft tissue algorithm, mediastinal window: 400 to 500HU window width, 40 to 50HU window position. All parameters were acquired from CT plain scan images.

### Image analysis

**Fig. 2:**
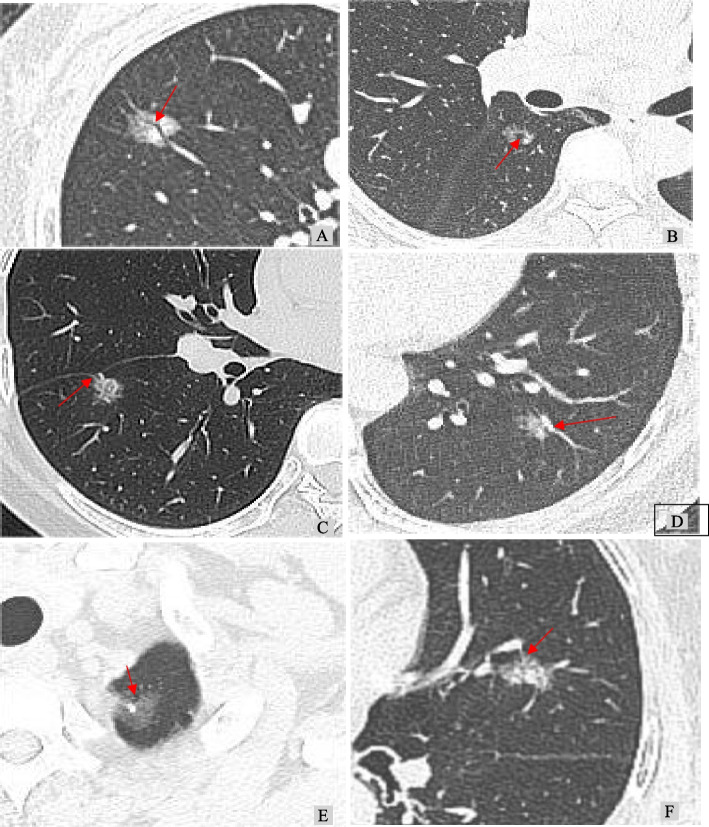
**A** Air bronchogram sign; **B** Vacuole sign; **C** Pleura traction sign; **D** Vascular convergence sign; **E** Lobulation sign; **F** Spiculation sign

Two chest radiologists, each with over 15 years of experience, independently analyzed the images without access to the patients' clinical data or pathological diagnoses. Any discrepancies in their results were resolved through discussion [16, 17]. The HRCT features, including continuous and categorical variables were browsed and analyzed on the Picture Archiving & Communication System (Fig. 2). (1) Spiculation sign: spinous protrusions appear at the edge of nodules, with dense brush or fine line protrusions into the surrounding lung parenchyma; (2) Lobulation sign: uneven arc profile at the edge of nodules, toothed, or altered in a wavy pattern; (3) Vacuole sign: presence of one or more air density shadows less than 5 mm in the nodules, with smooth edges; (4) Air bronchogram sign: tubular branching with air density of varying lengths, seen in successive adjacent planes; (5) Vascular convergence sign: the vessels adjacent to the nodules shift toward the lesion due to pulling, connecting with the lesion, or concentrating toward the lesion; (6) Pleura traction sign: linear or tentorial shadow between the lesion and the pleura, or a star-shaped shadow; (7) Consolidation/tumor ratio(CTR): ratio of the maximum diameter of the solid component on the lung window to the maximum diameter of the nodule; (8) Maximum diameter: maximum diameter of lesions shown on the axial CT images [18]; (9) Mean CT value: in the axial CT position, the large bronchi, blood vessels, and vacuoles/cavities in the lesion were avoided as far as possible at the maximum cross section of the glass nodule, A region of interest (ROI) cursor was used to record the CT value [19]; (10) Maximum CT value: the area with high lesion density was repeatedly measured to obtain the maximum value when the ROI area was 10 mm^2^.

### Histopathological evaluation

The surgically resected specimens underwent fixation in 10% formalin, paraffin embedding, microtome slicing, and staining with HE. All specimens were classified according to the criteria of the 2015 WHO Classification of Lung Tumors [20]. Lung adenocarcinomas were classified as AIS, MIA, and IA. The definition of AIS was an adenocarcinoma lesion less than 3 cm in diameter with a pure lepidic pattern. The MIA classification stipulated a predominant lepidic pattern with an invasive component of less than 5 mm. The IA type was further classified by the predominant pattern using comprehensive histological subtyping of lepidic, acinar, papillary, micropapillary, and solid. The percentage of each histological component was recorded in 5% increments, with the predominant pattern defined as the one with the largest percentage. Two pathologists, each with over 15 years of clinical experience in the Pathology Department of the Third Affiliated Hospital of Kunming Medical University.

### Imaging feature selection

Univariate analysis was performed on the imaging characteristics of the IAC group and NIAC group in the training group. Multivariate logistic regression analysis was implemented with variables with a *P*-value below 0.05 in the univariate analysis, and statistically significant imaging features were selected as independent predictors of IAC. The variance inflation factor (VIF) value was used to evaluate the degree of collinear interference between independent variables. The imaging features of ground glass nodules in both the training group and the validation group were compared by independent sample T-tests and chi-square tests.

### Model construction and performance assessment

Multivariate logistic regression models were used to determine the effects of multiple factors on a nomogram, and only the factors with a *p*-value less than 0.05 according to multivariate logistic regression analysis were incorporated into the nomogram. R software was employed for creating a nomogram model for the prediction of the invasiveness of GGNs. In both the training and validation groups, the differentiation of the models was assessed by AUC values, the calibration of the models was estimated by calibration curves, the nomogram goodness of fit was evaluated by Hosmer–Lemeshow tests, and the clinical usefulness of the models was appraised by decision analysis curves. The bootstrap method was utilized to self-sample 1000 times for internal validation.

### Statistical methods

When comparing the IAC and n-IAC groups, two independent sample t-tests were used to assess continuous variables conforming to a normal distribution. For non-normally distributed data, Mann–Whitney U tests were employed. Continuous variables included maximum diameter, CTR, maximum CT value, and mean CT value. Categorical variables, including spiculation signs, vascular convergence signs, pleura traction signs, air bronchogram signs, lobulation signs, and vacuole signs, were analyzed using the Pearson chi-square test. Binary logistic regression analysis was performed on continuous and categorical variables that were significantly different (*P* < 0.05) in the univariate analysis. A simple logistic regression model was created using the backward elimination process. SPSS (version 26.0) and R software (version 4.2.1) were used for all the statistical analyses. The cutoff value was defined as the maximum value of Youden’s index. A *P* value < 0.05 was considered to indicate statistical significance.

## Results

### Clinical and pathological characteristics

Altogether, 702 patients with 748 GGNs were recruited in this research. Among them, 181 cases (25.8%) were male, 521 cases (74.2%) were female; 577 cases (82.2%) were < 60 years old and 125 cases (17.8%) were ≥ 60 years old. There were 245 GGNs in the IAC group, 310 in the non-IAC group (AIS: *n* = 81; MIA: *n* = 229) in the training group; there were 93 GGNs in the IAC group, and 102 in the NIAC group (AIS: *n* = 9; MIA: *n* = 93) in the validation group. Table 1 exhibits the detailed clinical data of patients.

**Table 1:** General data results of patients

Variables	Total	training group	validation group
	(*n* = 702)	(*n* = 510)	(*n* = 192)
Sex/No.(%)			
male	181 (25.8)	144 (28.2)	37 (19.3)
female	521 (74.2)	366 (71.8)	155 (80.7)
Smoking/No.(%)			
Former/Current	133 (18.9)	116 (22.7)	34 (17.7)
Never	569 (81.1)	394 (77.3)	158 (82.3)
Age/No.(%)			
< 60	577 (82.2)	410 (80.4)	167 (87.0)
≥ 60	125 (17.8)	100 (19.6)	25 (13.0)
Nodule characteristics^*^			
pGGN	217 (29)	165 (29.7)	52 (26.9)
PSN	531 (71)	390 (70.3)	141 (73.1)
Location^*^(%)			
Right upper lobe	213 (28.5)	157 (28.3)	56 (29.0)
Right middle lobe	46 (6.2)	38 (6.8)	8 (4.2)
Right lower lobe	152 (20.3)	111 (20.0)	41 (21.2)
Left upper lobe	209 (27.9)	158 (28.5)	51 (26.4)
Left lower lobe	128 (17.1)	91 (16.4)	37 (19.2)
Pathological subtype^*^(%)			
AIS	90 (12)	81 (14.6)	9 (4.7)
MIA	322 (43)	229 (41.3)	93 (48.2)
IAC	336 (45)	245 (44.1)	91 (47.1)

^*^Total nodes = 748; nodes in training group = 555; nodes in validation group = 193

### Analysis and selection of imaging features

In the training group, the univariate analysis signified that multiple imaging features such as maximum diameter (12 vs. 9 mm, *P* < 0.001), CTR (40% vs.5%, *P* < 0.001), mean CT value (-370 vs. -540HU,* P* < 0.001), and maximum CT value (-12 vs. -280 HU, *P* < 0.001) were larger in the IAC group in comparison to the non-IAC group (Table 2). There were more pleura traction sign, vascular convergence sign, spiculation sign, and lobulation sign in the IAC group (*P* < 0.001) (Table 2).

**Table 2:** Relationship between CT features and invasiveness of ground glass nodules

CT feature	NIAC (*n* = 310)	IAC (*n* = 245)	*P*
maximum diameter(cm)	0.9 (0.7, 1.2)	1.2 (1, 1.5)	< 0.001
CTR(%)	5 (0, 20)	40 (30, 60)	< 0.001
maximum CT value(HU)	-280 (-380, -220)	-12 (-162, 110)	< 0.001
mean CT value(HU)	-540 (-629.75, -450)	-370 (-460, -275)	< 0.001
spiculation sign(%)			< 0.001
No	268 (86.45)	105 (42.86)	
Yes	42 (13.55)	140 (57.14)	
vascular convergence sign(%)			< 0.001
No	273 (88.06)	69 (28.16)	
Yes	37 (11.94)	176 (71.84)	
Pleura traction sign(%)			< 0.001
No	277 (89.35)	177 (72.24)	
Yes	33 (10.65)	68 (27.76)	
Air Bronchogram Sign(%)			0.151
No	301 (97.1)	231 (94.29)	
Yes	9 (2.9)	14 (5.71)	
lobulation sign(%)			< 0.001
No	282 (90.97)	179 (73.06)	
Yes	28 (9.03)	66 (26.94)	
Vacuole Sign(%)			0.133
No	228 (73.55)	165 (67.35)	
Yes	82 (26.45)	80 (32.65)	

Binary logistic regression analysis disclosed that the maximum diameter (OR = 4.707, 95%CI: 2.06–10.758), CTR (OR = 1.027, 95%CI: 1.011–1.043), maximum CT value (O R = 1.025, 95%CI: 1.004–1.047), mean CT value (OR = 1.035, 95%CI: 1.008–1.063), spiculation sign (OR = 2.055, 95%CI: 1.148–3.679), and vascular convergence sign (OR = 2.508, 95%CI: 1.345–4.676) were validated to be independent risk parameters for IAC of GGN (*P* < 0.05) (Table 3). The collinearity test results of the 6 independent risk parameters uncovered that there was no collinearity relationship between the 6 independent variables. Meanwhile, the ROC curves of the 6 independent risk factors were plotted. According to the Yoden index, the optimal cutoff values of maximum diameter, CTR, maximum CT value, and mean CT value were 9.5 mm, 23.5%, -139.5HU, and -495HU, respectively. No significant difference was observed in CT features between the training and validation groups, implying that they could be used as the training and validation groups (Table 4).

**Table 3:** Multivariable Logistic Regression of CT finding and invasiveness of ground glass nodules

	Univariate		Multivariate	
	OR (95% CI)	*P *	OR (95% CI)	*P *
maximum diameter	9.348 (5.346-16.345)	＜0.001	4.707 (2.060-10.758)	0.001
CTR	1.074 (1.062-1.086)	＜0.001	1.027 (1.011-1.043)	0.001
maximum CT value	1.095 (1.079-1.112)	＜0.001	1.025 (1.004-1.047)	0.019
mean CT value	1.122 (1.100-1.144)	＜0.001	1.035 (1.008-1.063)	0.012
spiculation sign		＜0.001	2.055 (1.148-3.679)	0.015
No	Reference			
Yes	8.508 (5.634-12.847)			
vascular convergence sign		＜0.001	2.508 (1.345-4.676)	0.004
No	Reference			
Yes	18.820 (12.096-29.282)			
Pleura traction sign		＜0.001	1.174 (0.595-2.316)	0.644
No	Reference			
Yes	3.225 (2.043-5.091)			
Air Bronchogram Sign		0.105		
No	Reference			
Yes	2.027 (0.862-4.765)			
lobulation sign		＜0.001	1.615 (0.822-3.175)	0.164
No	Reference			
Yes	3.713 (2.298-6.002)			
Vacuole Sign		0.111		
No	Reference			
Yes	1.348 (0.934-1.947)

**Table 4:** Comparison of CT features between the training group and the validation group

CT features		training group	validation group	*P*
		555	193	
maximum diameter(cm)		1.08 ± 0.34	1.03 ± 0.33	0.077
CTR(%)		25.98 ± 26.00	27.08 ± 25.39	0.612
maximum CT value(HU)		183 ± 209.15	185.81 ± 202.81	0.904
mean CT value(HU)		466.13 ± 146.13	449.69 ± 122.85	0.162
spiculation sign(%)	No	373 (67.2)	122 (63.2)	0.312
	Yes	182 (32.8)	71 (36.8)	
Pleura traction sign(%)	No	454 (81.8)	162 (83.9)	0.503
	Yes	101 (18.2)	31 (16.1)	
vascular convergence sign(%)	No	342 (61.6)	117 (60.6)	0.806
	Yes	213 (38.4)	76 (39.4)	
Air Bronchogram Sign(%)	No	532 (95.9)	187 (96.9)	0.521
	Yes	23 (4.1)	6 (3.1)	
lobulation sign(%)	No	461 (83.1)	161 (83.4)	0.909
	Yes	94 (16.9)	32 (16.6)	
Vacuole Sign(%)	No	393 (70.8)	147 (76.2)	0.153
	Yes	162 (29.2)	46 (23.8)	

### Construction and validation of nomogram models

**Fig. 3:**
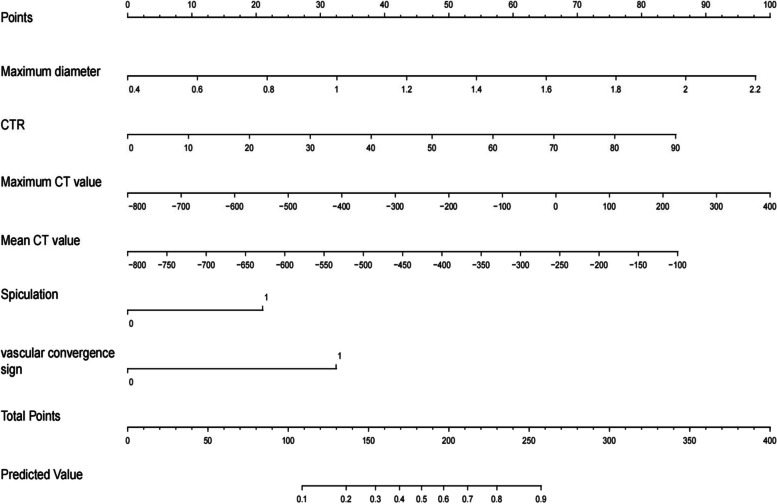
A nomogram model predicting the occurrence of IAC in GGN patients

**Fig. 4:**
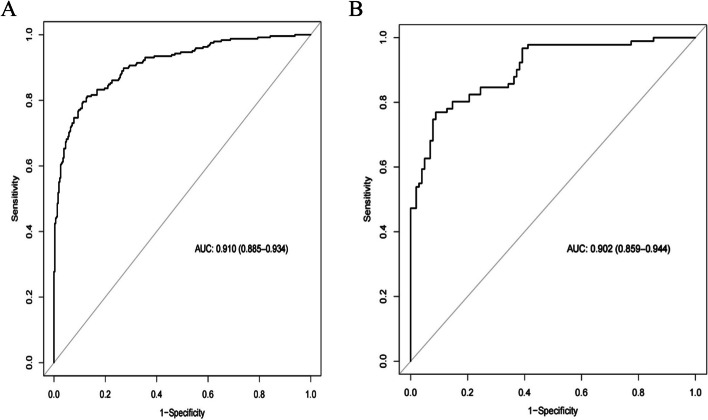
**A** ROC curve of the nomogram in training group. **B** ROC curve of the nomogram in validation group

**Fig. 5:**
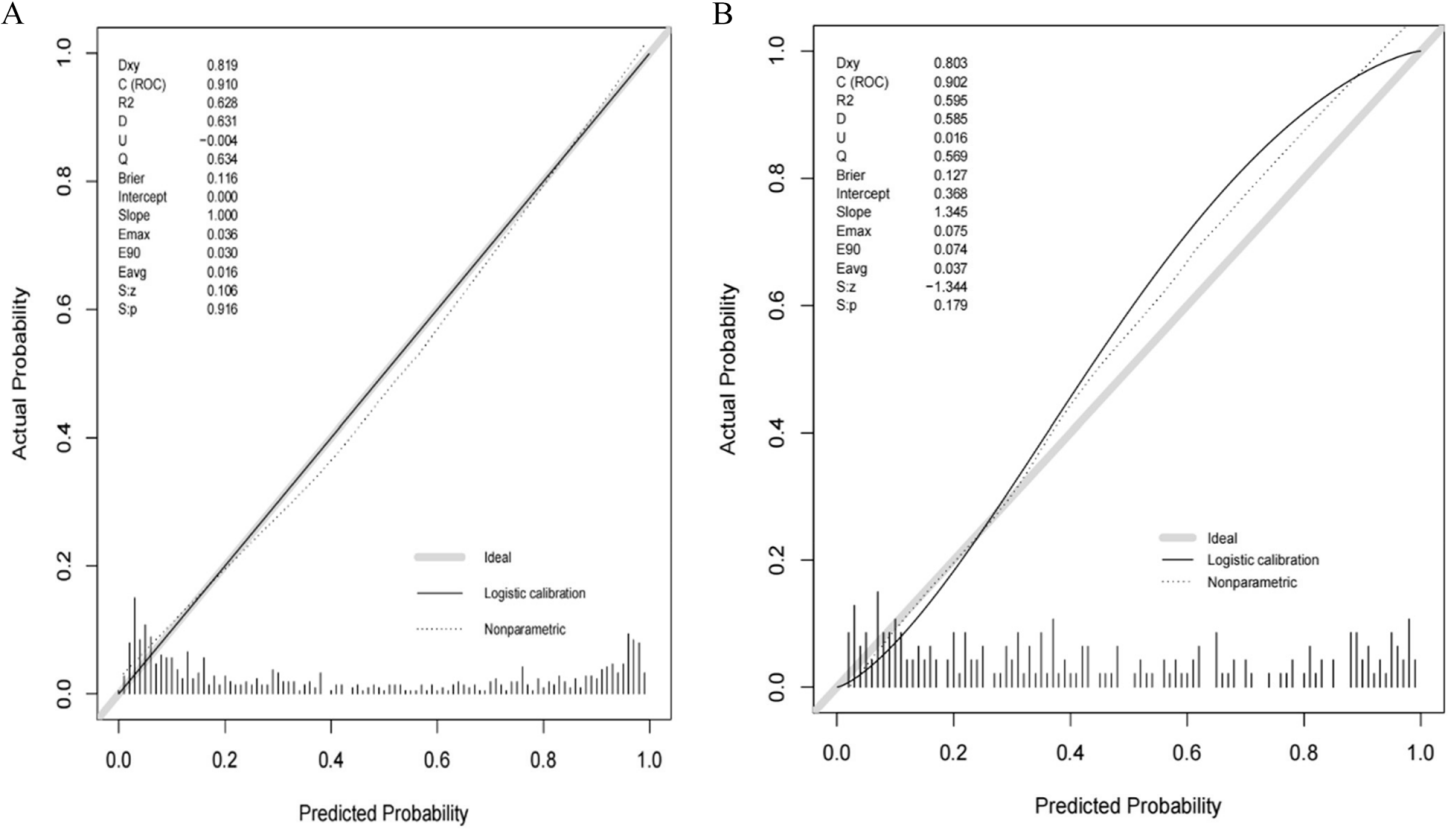
**A** Calibration curve of the nomogram in training group. **B** Calibration curve of the nomogram in validation group

**Fig. 6:**
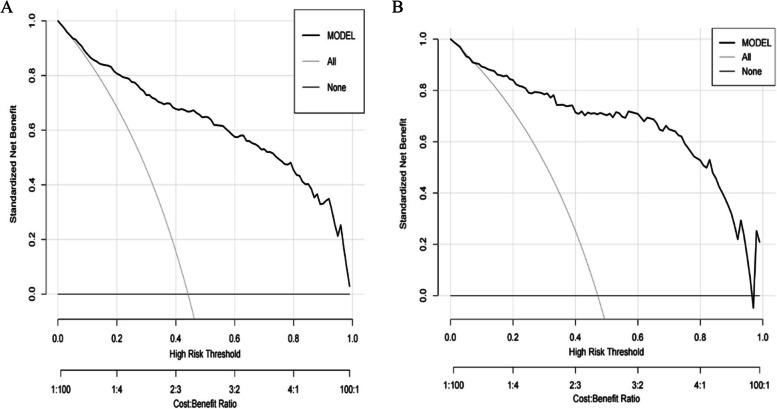
**A** Decision curve analysis of the nomogram in training group. **B** Decision curve analysis of the nomogram in validation group

Based on the maximum diameter, CTR, maximum CT value, mean CT value, spiculation sign, and vascular convergence sign, we created a nomogram model for predicting the invasiveness of GGNs (Fig. 3). The values of AUC were 0.910 (95%CI: 0.885–0.934) and 0.902 (95%CI: 0.859–0.944) in the training group and validation group, respectively, suggesting that the model was well differentiated (Fig. 4). Additionally, the Hosmer–Lemeshow goodness of fit test of both the training and validation groups suggested that the model had a good fitting effect (*P* > 0.05). Meanwhile, the calibration curve of the nomogram model unveiled good agreement between prediction and observation in both groups (Fig. 5). The decision analysis curves of the two groups indicated that the model had good clinical practicability (Fig. 6). Moreover, the AUC value of the Bootstrap internal verification method was 0.905, indicating that the model still had a high differentiation ability in the internal verification.

## Discussion

Lung cancer is the main malignant tumor that threatens human health, and adenocarcinoma is its longest histological subtype [21]. With the popularization of lung cancer screening, more and more lung adenocarcinoma with GGNs has been discovered [22]. At present, the management strategy of GGNs is still controversial. Inappropriate management of GGNs is very common in clinical practice. For AIS and MIA, regular follow-up is advisable to choose the appropriate operative time, and the surgery should be simplified to minimize surgical trauma [23]. For IAC, aggressive surgical treatment is required [24]. Therefore, accurate identification of IAC in GGNs is helpful for clinical decision-making. Other studies have shown [23] that for AIS and MIA, ensuring that the incisal margin is 5 mm away from the lesion is sufficient for clinical treatment, while for IAC, a stereoscopic incisal margin of 2 cm is required [24]. Moreover, there are differences in lymph node processing between NIAC and IAC patients [25]. For AIS and MIA, lymph node processing is not required, however in IAC, lymph node dissection or sampling is needed. Accurate identification of the two markers is helpful for selecting the appropriate surgical methods. Our results demonstrated that maximum diameter, CTR, maximum CT value, mean CT value, spiculation sign, and vascular cluster sign were independent risk factors for invasiveness of GGN. Based on this, the nomogram model for predicting the invasiveness of GGN could be used to identify IAC and provide a basis for clinical management decisions of GGNs.

Maximum diameter is an important parameter to evaluate the infiltration of GGN. Zhang et al. [26] included 124 patients in their study and found that the maximum diameter of GGN was a predictor of the invasiveness of GGN, with the highest diagnostic value at a critical value of 10 mm. The study by Fu et al. [7] showed that the maximum diameter could be used to identify the invasion of GGN, with the highest diagnostic performance at the diameter of 1.05 cm. Our results are consistent with these views and have discriminative implications for IAC when the cut-off value of the maximum diameter is 9.5 mm. The optimal diagnostic threshold value obtained by some scholars is larger than that proposed by us [27, 28]. This may be due to the fact that the nodules in their study were larger than those we included.

The solid component of GGN may reflect the degree of invasion. Currently, the measurement standard of the solid component of GGN is still controversial. Some scholars have shown that [29, 30] compared with the mediastinal window, solid components on the lung window are closely related to the degree of invasion of GGN. At present, most scholars use the solid part of the lung window for their research. CTR is a commonly used imaging index to evaluate the invasiveness of GGNs in the clinic. The results of the Japan Clinical Oncology Group (JCOG 0201) study showed [31] that the diagnostic criterion for IAC was CTR ≤ 0.5. This was similar to the results obtained by Shinya Katsumata et al. [32] in a retrospective study involving 744 patients. Our results suggested that CTR was an independent predictor of IAC, and there was a higher likelihood of IAC when the CTR exceeded 23.5. This difference between the two studies may be caused by different definitions of outcome variables. The NIAC in the two studies was defined as lung adenocarcinoma pathologically without lymph node invasion and vascular invasion, which also included a portion of the defined IAC in our paper, making the critical diagnostic value larger than ours. As part of alveolar collapse, changes in inflammation and fibrosis also appear in the form of high density [33], at this time, the judgment of the infiltration of GGN by CTR alone shows its limitations, and the diagnosis accuracy can be improved by combining other imaging features. Clear separation of solid components, small nodular lesions, concentrated distribution of solid components, and uniform density of solid components are predictors of non-invasive lesions with solid components [34].

The mean CT value of GGNs was mostly determined by the degree of myofibroblast matrix thickening caused by tumor cells invading normal lung tissues, and the higher infiltration ability corresponded to the higher mean CT value. Kitami et al. [35] found that the mean CT value had limited diagnostic ability for IAC in a study involving 78 GGNs. Subsequently, Zheng et al. [36] included 288 patients in their study and indicated that GGNs with a mean CT value of more than − 449.5 HU tended to be IAC. Another meta-analysis included articles published as of March 20, 2020, which showed that the mean CT value had a good discriminating effect on the invasiveness of GGNs with a sensitivity of 0.78 and specificity of 0.81 when the mean CT value was > -484HU [11]. These studies are similar to our results, which signifies that the mean CT value is an independent predictor of IAC and the efficacy of diagnosing IAC is greatest when the mean CT value is > -495HU. However, Fu et al. [7] proposed that the mean CT value could not be employed to indicate IAC. In this study, the subjects all had pGGNs with small changes in their mean CT values, which may be the reason for the non-significant differences between the groups.

Ichinose et al. [12] showed that the maximum CT value could identify invasive lesions (including MIA/IAC) in pGGN when the maximum CT value was ≥ -300 HU. Different density ranges and thresholds are able to distinguish different pathologic types, and the diagnostic threshold at IAC identification is larger than that obtained by Ichinose et al. Yue et al. [37] pointed out that with a cut-off value of -107HU for the maximum CT value, the sensitivity and specificity of IAC diagnosis were 92% and 77%, respectively. This is consistent with our results that GGN is more likely to be IAC when the maximum CT value is > -139.5HU. This may be caused by the increased degree of tumor invasion, the replacement of the majority of normal lung tissue by cancer cells, and massive accumulation and deep infiltration of tumor cells.

Our results indicated that the spiculation sign was an independent predictor of IAC, which was the same as the results of Si [18]. This phenomenon may be caused by the increased invasion of IAC, the increased number of tumor cells, and the infiltration into surrounding tissues. Vascular convergence sign was also an independent predictor of IAC, which was the same as the study results of Gao et al. [38], and Zhang et al. [39] also came to a similar conclusion after extensive research, proposing a new model of "ground glass nodules tumor microangiography sign" for early diagnosis of lung cancer. The reasons for this phenomenon may be explained as follows: first, the increase in infiltration will lead to an increased in oxygen consumption, which will affect the blood supply vessels and lead to an increase in permeability and diameter. Second, an increase in the invasion degree will increase in fibrosis focus, further leading to blood vessel aggregation around the tumor [40].

Compared with the ordinary correlation analysis, the nomogram prediction model can systematically integrate the relevant features of the research object and jointly apply them to maximize the prediction efficiency. The nomogram is a way of presenting the prediction model that can make the model visualized, personalized, and convenient for clinical use [41]. In recent years, for the prediction of the invasiveness of GGN, some scholars have established a nomogram model based on radiomics characteristics, which improves the diagnostic ability, but also restricts the clinical application to a certain extent [42–44]. Other scholars [13–15, 45] identified the invasiveness of GGN by using simple and easily available imaging features, and its diagnostic efficacy was also improved. However, most of these studies focused on judging the invasion of pGGN [15, 45]. The rare prediction model of mGGN did not systematically evaluate the CT imaging features of GGN and included small sample size, and the model was not verified [13]. Clinically, mGGN accounts for a large part of GGN [46]. To enhance the widespread use of the model in clinical practice, we included both pGGN and mGGN in our paper, and CTR was first introduced into the construction of the model, followed by evaluating and verifying the model. Our results suggested that the model constructed by comprehensive use of CT imaging features showed good differentiation and calibration abilities, and had good clinical practicability in the training and validation groups. In comparison to previous studies, this was the first time to systematically explore the relationship between the quantitative and qualitative features of CT imaging and the invasiveness of GGN on the basis of large samples, and to construct a prediction model with good prediction ability.

Although the conclusion is encouraging, some limitations should still be discussed. The research population recruited in this paper were all patients from our center, which was a single-center retrospective study with a narrow study population. Despite the performance of the external validation of our center at different periods, there was still a lack of multi-center and prospective data to further verify the results. The imaging features of this study were artificial measurements, which inevitably led to certain measurement errors. Although we systematically included the imaging features of GGN, however, imaging data for long-term observation of the GGN course is lacking due to the long course of GGN. If follow-up imaging data are available, the follow-up time of GGN can be added to the model as a variable. Due to the early screening of lung cancer, ground glass nodules were first found earlier, which made the nodules included in this study more concentrated on small nodules (< 2.2 cm). When the nodules were > 2.2 cm, the prediction efficiency of the prediction model constructed was limited. In future studies, we hope to expand the dataset to include nodules with larger diameters to refine the model and increase its availability in the clinic. Additionally, there are no uniform acceptance criteria for the performance of nomograms, and the impact of nomograms on clinical decision-making and patient satisfaction is unclear.

## Conclusion

Combined with the quantitative and qualitative features of CT imaging, a nomogram prediction model can be established to predict the infiltration degree of GGNs. This model has a good prediction effect on the infiltration degree of GGN and can provide help for the clinical management and decision-making of GGN.

## Data Availability

The datasets generated and/or analyzed during the current study are not publicly available due sharing data is not included in our research institution review board but are available from the corresponding author on reasonable request.
